# Defining and Measuring Engagement and Adherence in Digital Mental Health Interventions: Protocol for an Umbrella Review

**DOI:** 10.2196/73438

**Published:** 2025-07-28

**Authors:** Lyen Krenz Yap, Edel Ennis, Maurice Mulvenna, Jorge Martinez-Carracedo

**Affiliations:** 1 School of Psychology Ulster University Coleraine United Kingdom; 2 School of Computing Ulster University Belfast United Kingdom

**Keywords:** engagement, adherence, user retention, digital mental health interventions, umbrella review, systematic review, mHealth, eHealth

## Abstract

**Background:**

Digital mental health interventions (DMHIs) offer scalable solutions to address mental health needs, particularly among marginalized populations. However, engagement and adherence rates in DMHIs are often suboptimal, limiting their potential impact. Despite the growing body of literature on DMHI engagement, there is no consensus on how engagement and adherence are defined and measured across studies. Understanding these variations is crucial to improving DMHI design, evaluation, and outcomes.

**Objective:**

Using the population, concept, context framework to frame the objectives, this umbrella review aims to synthesize existing systematic reviews, meta-analyses, and scoping reviews to identify how engagement and adherence are defined and measured in DMHIs. Additionally, this review seeks to explore factors that may influence DMHI engagement and adherence.

**Methods:**

A systematic search of peer-reviewed literature will be conducted across major electronic databases following PRISMA (Preferred Reporting Items for Systematic Reviews and Meta-Analyses) guidelines. Eligible studies will include systematic reviews, meta-analyses, and scoping reviews published in English in the past 10 years that examine engagement and/or adherence in DMHIs. Data will be extracted and synthesized to identify definitions, measurement methods, and influencing factors. Risk of bias will be assessed using the Joanna Briggs Institute (JBI) critical appraisal checklist for systematic reviews and research syntheses. Findings will be presented using a mixed methods convergent integrated approach, identifying and synthesizing themes across the included quantitative and qualitative study results.

**Results:**

This study is expected to be conducted over a 6-month period. The search, conducted in early March 2025, initially identified 5087 papers. An additional 35 papers were found through manual handsearching of BMC Digital Health. These totals were recorded prior to the removal of duplicates. This umbrella review is expected to be conducted with screening, quality assessment, and data extraction streamlined through the Covidence platform. The screening and selection of studies will be performed in month 1, followed by data extraction and quality appraisal in months 2 and 3. Data synthesis and integration will take place in months 4 and 5, and writing conclusions and preparing the manuscript will occur in month 6. This review will provide a comprehensive summary of how engagement and adherence are operationalized across existing literature. It will highlight commonalities, inconsistencies, and gaps in definitions and measurement methods. Additionally, this review will outline the key factors that influence engagement and adherence, including individual, technological, and contextual elements.

**Conclusions:**

This umbrella review will contribute to a more nuanced understanding of engagement and adherence in DMHIs, informing future intervention design and evaluation. The findings will support the development of standardized definitions and measurement frameworks, ultimately enhancing the effectiveness and inclusivity of DMHIs.

**Trial Registration:**

PROSPERO CRD42025637603; https://www.crd.york.ac.uk/PROSPERO/view/CRD42025637603

**International Registered Report Identifier (IRRID):**

PRR1-10.2196/73438

## Introduction

Digital mental health interventions (DMHIs) are technology-enabled tools that deliver psychological information, support, or therapy through platforms such as mobile apps, websites, and telehealth services. These interventions aim to promote mental well-being and help users manage mental health conditions [1,2]. By offering flexibility, cost-effectiveness, and accessibility, DMHIs provide important alternatives to traditional face-to-face services, particularly for individuals who encounter barriers to in-person care [3,4]. As the demand for scalable mental health solutions grows globally, DMHIs have become central to contemporary public health strategies. However, despite their promise, DMHIs frequently suffer from low engagement and poor adherence, which limit their long-term effectiveness and scalability [5,6]. Many users discontinue use prematurely or do not engage with the content in a sustained or meaningful way. A major barrier to resolving these challenges is the lack of agreement on how engagement and adherence are defined and measured in the literature.

Some studies focus on narrow behavioral metrics such as login frequency or total time spent [7], while others consider broader outcomes such as initial uptake, continued use, or program completion rates [6]. In some cases, superficial indicators such as app downloads are mistakenly equated with meaningful engagement, despite offering little insight into user experience or therapeutic benefit [8]. Recent studies have advocated for a more comprehensive understanding of engagement that includes cognitive (eg, attention, comprehension) and emotional (eg, motivation, satisfaction) dimensions alongside behavioral indicators. This broader perspective emphasizes the quality of user interaction with DMHIs, rather than solely the quantity [9-11].

Current definitions and measurement approaches remain highly inconsistent. This heterogeneity poses significant challenges to the comparability of findings, the development of standardized evaluation frameworks, and the creation of effective, evidence-based strategies for improving user engagement and retention. Addressing this issue is essential for advancing the design, implementation, and evaluation of DMHIs. Without conceptual and methodological clarity, it is difficult to determine which interventions are effective, under what conditions, and for whom.

This umbrella review aims to synthesize existing systematic reviews, meta-analyses, and scoping reviews to provide a comprehensive understanding of the definitions, measurement approaches, and factors that influence engagement and adherence in DMHIs. To frame the review’s objectives, the population, concept, context framework recommended by Joanna Briggs Institute (JBI) is applied, as outlined in Table 1. This umbrella review aims to examine how engagement and adherence is defined and measured in systematic reviews, meta-analyses, and scoping reviews of DMHIs. This review also investigates whether there are other factors that influence DMHI engagement and adherence.

**Table 1:** Objectives of the umbrella review.

JBI^a^ framework	Measurements
Population	All populations (no restrictions)
Concept	Definition and measurement of engagement and adherence
Context	Digital mental health interventions

^a^JBI: Joanna Briggs Institute.

## Methods

### Database Selection

No ethics approval will be required, as this review is based on already published and publicly available data. The databases utilized for this umbrella review include Web of Science, CINAHL via EBSCO, MEDLINE via Ovid, PsycINFO via Ovid, Cochrane via EBM Reviews, and ProQuest Complete. Full access to these databases is provided by the Ulster University library. An additional handsearching of the BMC Digital Health journal will also be conducted. These databases were selected for their comprehensive coverage of peer-reviewed literature in relevant subject matters of psychology, health care, and digital health interventions [12,13].

From the set of key publications identified by the authors, there was one paper [7] from BMC Digital Health. As this journal is relatively new and has not yet been indexed in major databases, there was a risk of missing additional relevant studies. After discussion with the review team and to ensure the comprehensiveness of the search, it was decided to include BMC Digital Health through handsearching, a method where journals are manually browsed to identify potentially relevant studies that may not be captured through database searches [14].

In addition to this, CINAHL and Cochrane did not yield the expected relevant studies from this set of key publications. Therefore, precision testing and unique hits testing were conducted on CINAHL to assess the database’s contribution to the overall search strategy [15]. The results indicated that CINAHL contributed a small number of unique relevant records not identified in other databases, thereby justifying its inclusion for comprehensiveness. Cochrane, on the other hand, was retained due to its established role as a specialized database for high-quality systematic reviews and evidence syntheses, particularly within health and clinical research [16].

### Search Strategy

Database searches, with search strategies developed in assistance with the subject librarian, will include the following concepts: engagement, adherence, digital (web-based, smartphone, app, computer), mental health (well-being, emotional health, anxiety, stress), and interventions (promotion, prevention, treatment). The search will focus on identifying systematic reviews, meta-analyses, and scoping reviews that were peer-reviewed and published between 2015 and 2025 in English (Multimedia Appendix 1).

A combination of keyword searching and subject heading searching will be employed using the appropriate Boolean operators. Keyword searching identifies studies based on specific words appearing in titles, abstracts, or full texts, while subject heading searching uses controlled vocabulary to capture relevant studies, regardless of terminology variations. Combining both approaches enhances search sensitivity and specificity. Gray literature will be excluded, but supplementary searching using forward citation-chaining using the citation chaser tool will be utilized to ensure that the reviews included are up to date [17]. To ensure comprehensiveness and accuracy, searches will be rerun prior to the analysis stage.

Efforts were made to validate the search strategy. Following guidance from the Cochrane Handbook [18], the team tested whether the search strategy could retrieve key publications with which they were already familiar. To mitigate bias, preliminary citation chaining was also conducted to identify additional relevant studies that may not have been known in advance. Matters regarding databases are discussed above.

### Eligibility Criteria

Search criteria already structure studies that are eligible for inclusion. Within the search results, the Population, Intervention, Comparator, Outcome, and Study Design framework is applied to guide the inclusion and exclusion of studies. This will be added on the Covidence platform for reference when examining eligibility. This is elaborated in Table 2. The included studies focus on DMHIs that define or measure engagement and/or adherence. Eligible study designs include systematic reviews, meta-analyses, and scoping reviews that examine engagement and adherence in DMHIs. Studies will be excluded if they evaluate digital interventions without a mental health outcome, focus on non-DMHIs, or discuss digital mental health without an intervention component. Additionally, reviews that do not sufficiently define or measure engagement and adherence as well as noneligible study designs such as integrative reviews, narrative reviews, primary studies, or research protocols will be excluded. No restrictions are applied to comparators or populations.

**Table 2:** Inclusion and exclusion criteria.

	Inclusion criteria	Exclusion criteria
Population	No restrictions applied	No restrictions applied
Intervention	Digital mental health interventions	Digital interventions without mental health outcomes Mental health interventions delivered nondigitally Digital mental health papers without interventions
Comparator	Not applicable	Not applicable
Outcome	Definition or measurement of engagement; definition or measurement of adherence	No sufficient details on definition or measurement of engagement or adherence
Study design	Systematic reviews, meta-analysis, and scoping reviews	Any other type of review (including but not limited to integrative reviews, narrative reviews), independent studies, or research protocols

### Screening and Selection

The primary reviewer and a subject librarian will comprehensively search the relevant databases. After removing duplicates, the references will be imported into the Covidence platform for screening. Two independent reviewers will assess the titles and abstracts against the eligibility criteria, resolving disagreements through discussion. If necessary, a third reviewer will be consulted to achieve consensus. Full-text articles of potentially eligible studies will then be extracted, where possible, and reviewed independently by 2 reviewers for relevance and eligibility, with disagreements addressed in consultation with the project team. The reasons for study exclusion at both the abstract and full-text stages will be documented. Each step of the process will be tracked and reported using a PRISMA (Preferred Reporting Items for Systematic Reviews and Meta-Analyses) flowchart (Multimedia Appendix 2).

### Data Extraction

Once the citations to be included in the umbrella review are identified, data extraction will be conducted using Covidence’s Extraction 2 tool to ensure a structured and standardized approach. The following data will be extracted: references (authors, year of publication), study design (systematic review, meta-analysis, or scoping review), research aims, data synthesis approach, type and number of studies included, demographic information, description of DMHIs, outcome variables, and main findings. To assess the quality of their primary data, their overall critical appraisal scores and quality assessment indicators will also be extracted. This information will be organized into data extraction tables on review characteristics, included study characteristics, participant characteristics, and study results. Covidence will facilitate consistency across reviewers and minimize data entry errors. For missing or unreported data, if deemed necessary, the original authors of the study will be contacted. All findings will be reported following the PRISMA 2020 guidelines and its extension for umbrella reviews.

### Quality Assessment

The methodological quality of the included systematic reviews, meta-analyses, and scoping reviews will be evaluated using the JBI critical appraisal checklist for systematic reviews and research syntheses. Utilizing the Covidence platform, 2 reviewers will independently assess the selected studies, resolving any disagreements through detailed discussions. If required, a third reviewer will be involved to reach a consensus.

Evaluating overlap is critical, as it can lead to the disproportionate weighting of certain primary studies within the analysis. Given that no widely accepted method currently exists for handling overlap in umbrella reviews [19], this study will consider the approach of similar reviews, the scope of the review question, and the breadth and complexity of the final set of the included studies when considering how best to manage overlap. A dedicated section in the discussion will address how duplication of primary studies was handled in the overall analysis.

### Data Synthesis

Due to the presumed heterogeneity of the data, the data synthesis will follow a mixed methods convergent integrated approach. Quantitative data on engagement and adherence metrics (eg, usage frequency, completion rates, adherence duration) will be synthesized descriptively. Concurrently, qualitative data such as narrative definitions, thematic insights, and contextual factors influencing engagement and adherence will undergo thematic analysis to identify patterns and broader themes. Both data types will be synthesized and reported side by side, ensuring that the insights from each complement and enhance the understanding of how engagement and adherence are defined, measured, and influenced in DMHIs. Study conclusions will also draw on the study’s implications for coproduction, patterns of engagement, and inclusivity in DMHIs.

## Results

The search, conducted in early March 2025, initially identified 5087 papers. An additional 35 papers were found through manual handsearching of BMC Digital Health. These totals were recorded prior to the removal of duplicates. This umbrella review is expected to be conducted over a 6-month period, with screening, quality assessment, and data extraction streamlined through the Covidence platform. The screening and selection of studies will be performed in month 1, followed by data extraction and quality appraisal in months 2 and 3. Data synthesis and integration will take place in months 4 and 5, and writing conclusions and preparing the manuscript will occur in month 6. Regular weekly meetings among the whole review team will be conducted to monitor progress and ensure timely completion of each phase.

The results will be presented in terms of the definitions of “engagement” and “adherence” within the context of digital health, along with the qualitative and quantitative methods and tools used to measure these concepts in DMHIs. Additionally, the review will outline key factors that influence engagement and adherence, including individual elements such as age, gender, mental health status, and digital literacy; technological factors such as intervention design features, usability, and personalization; and contextual aspects such as social support, accessibility, and the broader health care environment. The exploration of these factors will provide a comprehensive understanding of the complex and dynamic nature of engagement and adherence in DMHIs.

## Discussion

### Principal Findings

Given the inconsistencies in how engagement and adherence are defined and measured in DMHIs, this umbrella review is crucial in providing clarity and enabling meaningful comparisons across studies. The definitions of these constructs are often heavily influenced by the parameters of individual studies, and it is anticipated that they may differ depending on whether they are applied at the intervention level or the treatment level. For instance, engagement might be measured by the number of sessions attended within a study protocol versus actual participation in the intervention program itself. Additionally, related constructs such as attrition, dropout, adoption, and retention are often used interchangeably or inconsistently, further complicating synthesis efforts.

This review also highlights the potential importance of distinguishing between the quantity and quality of engagement, recognizing that engagement is not merely a behavioral metric but also involves emotional and cognitive investment. These conceptual nuances have significant implications for the design and reporting of DMHI studies. By addressing the use of umbrella terms and the tendency of existing reviews to focus solely on individual-level data, this study aims to promote more precise terminology and improve the interpretability and utility of DMHI studies. This review will outline future research priorities and needs for effectively understanding engagement and retention, as these are fundamental to the successful design, monitoring, and evaluation of DMHIs.

### Comparison With Prior Work

Although a select few reviews [5,7] have explored engagement and retention in digital interventions, many of these lack methodological rigor, often relying solely on narrative reviews, limited systematic searches, or qualitative studies. Additionally, much of the existing literature focuses on digital health applications more broadly, rather than digital mental health specifically, which presents unique challenges and nuances [5,7]. Several studies have emphasized the importance of further investigating engagement as a complex and multifaceted phenomenon within digital mental health contexts [12,13]. Given the rapid evolution of technology and the increasing integration of artificial intelligence in digital interventions, there is a pressing need for a timely and methodologically robust review to synthesize current evidence and guide future research and practice.

### Limitations

Given the expected heterogeneity of findings, the authors anticipate challenges in consolidating the data not only due to variations in definitions and measurements but also because of the wide range of existing interventions and the diverse mental health concerns they address. This underscores the need for a robust and systematic approach to data extraction and categorization. Furthermore, considering the rapid pace at which research in this field evolves, it will be essential to strike a balance between comprehensive analysis and timeliness. We also acknowledge that due to our focus on peer-reviewed reviews with methodological rigor, some potentially relevant studies may have been excluded as a result of omitting gray literature.

### Implications and Conclusion

This review’s findings have important implications for both research and practice. For researchers, the synthesis will offer a foundational understanding of how engagement and adherence are conceptualized, informing future study designs and methodological approaches. For practitioners and app developers, this review will highlight key factors that promote sustained engagement, supporting the development of more user-centered interventions. These insights will ultimately aid in enhancing user retention, improving mental health outcomes, and contributing to the overall quality of DMHI research.

### Dissemination Plan

To maximize this review’s impact, the findings will be disseminated through publication in a peer-reviewed journal and presentation at high-level international conferences involving key stakeholders in digital health and mental health research. Any deviations from this protocol will be recorded and justified in the final version of the study.

## Appendix

Multimedia Appendix 1 Search strategy.

Multimedia Appendix 2 PRISMA-P (Preferred Reporting Items for Systematic Reviews and Meta-Analyses Protocols) checklist.

## Abbreviations

DMHI: digital mental health intervention
JBI: Joanna Briggs Institute
PRISMA: Preferred Reporting Items for Systematic Reviews and Meta-Analyses
