# Supplementary material for: Defining and Measuring Engagement and Adherence in Digital Mental Health Interventions: Protocol for an Umbrella Review
Source: JMIR Res Protoc. 2025 Jul 28;14:e73438. doi: 10.2196/73438 (PMC12340454; doi:10.2196/73438)
Supplement: Multimedia Appendix 1 [file resprot_v14i1e73438_app1.docx]

| Line | Search Terms |
| --- | --- |
| 1 | Engag* OR Participat* OR Interact* OR Involv* OR Adopt* OR Use* OR Usage OR “Log In” OR “Sign In” OR Download* |
| 2 | Adher* OR Compliance OR Compliant OR Complying OR Complied OR Complete OR Completion OR Finish OR Retention OR Retain* OR Commit* |
| 3 | 1 or 2 |
| 4 | digital* or mHealth or "m-health" or eHealth or "e-health" or "web-based" or "internet-based" or "mobile phone*" or "cell* phone*" or "smart phone*" or smartphone* or "mobile device*" or "text messag*" or "text-based" or SMS or app* or "tele-health" or telehealth or computer* or online or "electronic health" or telemedicine or "tele-medicine" |
| 5 | "mental health" or "well-being" or wellbeing or "psychological health" or "emotional health" or "behavioral health" or "mental difficult*" or "mental illness*" or "mental disorder*" or "mental disease*" or "mood disorder*" or depress* or anxiet* or anxious or suicid* or stress* or psychotherap* or psychiatr* |
| 6 | Interven* or promot* or prevent* or program* or support* or policy or policies or implement* or evaluat* or therap* or develop* or Treatment* or help* |
| 7 | where possible, combine 4 and 5 and 6 using proximity operators |
| 8 | review* or meta-analys* or metanalys* or meta analys* |
| 9 | 3 and 7 and 8 |
| 10 | Limit 9 to English language and yr=”2015-Current”) and Peer-Reviewed Journals |

*Table S1: Line by Line Search Strategy*

*Table S2: Database-specific Headings and Operators*

| Database | Operators Applied | MeSH Terms |
| --- | --- | --- |
| PsycINFO  via OVID | “ab.ti” (title or abstract)  “adj3” (adjacent to 2 words) | - Mental health/   **AND**   - exp Digital Mental Health Resources/ **OR** - exp Telemedicine/ **OR** - exp Digital Interventions/ **OR** - exp Online Therapy/ |
| Medline  via OVID | “ab.ti” (title or abstract)  “adj3” (adjacent to 2 words) | - Mental health/   **AND**   - exp Digital Mental Health Resources/ **OR** - exp Telemedicine/ **OR** - exp Digital Interventions/ **OR** - exp Online Therapy/ |
| Web of Science | “ab.ti” (title or abstract)  “NEAR/2” (close to 2 words) | Not applicable. |
| CINAHL  Via EBSCO | “ab.ti” (title or abstract)  “N2” (close to 2 words) | - Mental health/   **AND**   - Telemedicine **OR** - Mental Health Teletherapy **OR** - Telepsychiatry |
| Cochrane  via EBM Reviews | “ab.ti” (title or abstract)  “adj3” (adjacent to 2 words) | - Mental health/   **AND**   - exp Digital Mental Health Resources/ **OR** - exp Telemedicine/ **OR** - exp Digital Interventions/ **OR** - exp Online Therapy/ |
| ProQuest | “ab.ti” (title or abstract)  “Near/2” (close to 2 words) | Not applicable. |

*Table S3: Hand Searching*

| Engagement + Digital Mental Health Interventions + Review | Adherence + Digital Mental Health Interventions + Review |
| --- | --- |
| (Engag* OR Participat* OR Interact* OR Involv* OR Adopt* OR Use* OR Usage OR “Log In” OR “Sign In” OR Download* ) AND (digital* or mHealth or "m-health" or eHealth or "e-health" or "web-based" or "internet-based" or "mobile phone*" or "cell* phone*" or "smart phone*" or smartphone* or "mobile device*" or "text messag*" or "text-based" or SMS or app* or "tele-health" or telehealth or computer* or online or "electronic health" or telemedicine or "tele-medicine") AND ("mental health" or "well-being" or wellbeing or "psychological health" or "emotional health" or "behavioral health" or "mental difficult*" or "mental illness*" or "mental disorder*" or "mental disease*" or "mood disorder*" or depress* or anxiet* or anxious or suicid* or stress* or psychotherap* or psychiatr*) AND (Interven* or promot* or prevent* or program* or support* or policy or policies or implement* or evaluat* or therap* or develop* or Treatment* or help*) AND (review* or meta-analys* or metanalys* or meta analys*) | (Adher* OR Compliance OR Compliant OR Complying OR Complied OR Complete OR Completion OR Finish OR Retention OR Retain* OR Commit*) AND (digital* or mHealth or "m-health" or eHealth or "e-health" or "web-based" or "internet-based" or "mobile phone*" or "cell* phone*" or "smart phone*" or smartphone* or "mobile device*" or "text messag*" or "text-based" or SMS or app* or "tele-health" or telehealth or computer* or online or "electronic health" or telemedicine or "tele-medicine") AND ("mental health" or "well-being" or wellbeing or "psychological health" or "emotional health" or "behavioral health" or "mental difficult*" or "mental illness*" or "mental disorder*" or "mental disease*" or "mood disorder*" or depress* or anxiet* or anxious or suicid* or stress* or psychotherap* or psychiatr*) AND (Interven* or promot* or prevent* or program* or support* or policy or policies or implement* or evaluat* or therap* or develop* or Treatment* or help*) AND (review* or meta-analys* or metanalys* or meta analys*) |
